# Immigrant women’s experiences of postpartum depression in Canada: a protocol for systematic review using a narrative synthesis

**DOI:** 10.1186/2046-4053-2-65

**Published:** 2013-08-21

**Authors:** Gina MA Higginbottom, Myfanwy Morgan, Joyce O’Mahony, Yvonne Chiu, Deb Kocay, Mirande Alexandre, Joan Forgeron, Marilyn Young

**Affiliations:** 1 Faculty of Nursing, Edmonton Clinic Health Academy, University of Alberta, 11405 87 Avenue, Edmonton AB T6G 1C9, Canada; 2 Primary Care & Public Health Sciences London, King’s College London, London SE1 3QD, UK; 3 School of Nursing, Thompson Rivers University, Kamloops, BC, Canada; 4 Multicultural Health Brokers Co-operative, Edmonton AB T5H 2 M6, Canada; 5 Health Canada, Public Health Agency of Canada, Calgary, Alberta T2G 4X3, Canada; 6 Citizenship and Immigration Canada, New Multiculturalism Grants and Contributions Program, Canada Place, Edmonton AB T5J 4C3, Canada; 7 Alberta Health Services, Lois Hole Hospital for Women, Edmonton AB T5H 3V9, Canada; 8 Alberta Health Services, Prenatal and Postpartum Services, Public Health, Calgary, AB, Canada

**Keywords:** Narrative synthesis, Immigrant women, Postpartum depression, Maternity care experiences, Canada, Systematic review protocol

## Abstract

**Background:**

Literature documents that immigrant women in Canada have a higher prevalence of postpartum depression symptomatology than Canadian-born women. There exists a need to synthesize information on the contextual factors and social determinants of health that influence immigrant women’s reception of and behavior in accessing existing mental health services. Our research question is: what are the ethnoculturally defined patterns of help-seeking behaviors and decision-making and other predictive factors for therapeutic mental health care access and outcomes with respect to postpartum depression for immigrant women in Canada?

**Methods/design:**

Our synthesis incorporates a systematic review using narrative synthesis of reports (peer- and non-peer reviewed) of empirical research and aims to provide stakeholders with perspectives on postpartum mental health care services as experienced by immigrant women. To reach this goal we are using integrated knowledge translation, thus partnering with key stakeholders throughout the planning, implementation and dissemination stages to ensure topic relevancy and impact on future practice and policy. The search and selection strategies draw upon established systematic review methodologies as outlined by the Centre for Reviews and Dissemination and also incorporate guidelines for selection and appraisal of gray literature. Two search phases (a database and a gray literature phase) will identify literature for screening and final selection based on an inclusion/exclusion checklist. Quality appraisal will be performed using the tools produced by the Centre for Evidence Based Management. The narrative synthesis will be informed by Popay *et al*. (2006) framework using identified tools for each of its four elements. The integrated knowledge translation plan will ensure key messages are delivered in an audience-specific manner to optimize their impact on policy and practice change throughout health service, public health, immigration and community sectors.

**Discussion:**

The narrative synthesis methodology will facilitate understandings and acknowledgement of the broader influences of theoretical and contextual variables, such as race, gender, socio-economic status, pre-migration history and geographical location. Our review aims to have a substantive and sustainable impact on health outcomes, practice, programs and/or policy in the context of postpartum mental health of immigrant women. PROSPERO registration number CRD42012003020.

## Background

The diverse and multicultural nature of Canadian society and Canada’s statutory commitment to multiculturalism [1,2] means that the synthesis of knowledge related to immigrant experiences in seeking, accessing and receiving health care services is an urgent imperative to realize the population’s health and well-being. Failure to provide culturally competent health care may impact negatively on health care interactions and outcomes, ranging from simple miscommunication to more profound detriments to health [3,4]. The impact can be particularly evident for immigrant women during maternity [4-6], which can exacerbate their socioeconomic marginalization and subsequent vulnerability. Synthesized evidence is needed for strategic enhancements to current maternity care provision including professional development of health professionals to ensure culturally congruent and culturally safe care.

Two analyses of data from the Canadian Maternity Experiences Survey, conducted during the 2006 Canadian census, concluded that postpartum depression (PPD) symptomatology has a prevalence ratio of 2.42 (confidence interval (CI) 1.89 to 3.06) for recent immigrants as compared to Canadian-born women of European descent [7] and that interventions for PPD symptomatology should target immigrant and adolescent mothers due to these groups’ higher risk [8]. Moreover, the aforementioned level of increased risk may be conservative since other studies have found relative risks for depressive symptomatology of four-to-five times for refugee and immigrant women, respectively [9] and an odds ratio of 2.97 (CI 1.70 to 5.17) for major PPD [10]. These statistics urge more attention to the timely identification and appropriate treatment of PPD among immigrant women.

### Knowledge gap on ethnocultural factors of PPD

Current literature reviews reveal a knowledge gap regarding the impact of ethnocultural factors on maternal depression [11,12]. Many studies have focused attention on how health care practices based on Western cultural concepts influence the ways in which immigrant women use mental health care services. Others have examined immigrant women’s perspectives about their social support preferences, the barriers they experience and their preferred support interventions [13,14]; moreover, difficulties may arise in relation to access to services and patterns of help-seeking [15], including language barriers, stigma-related concerns and discriminatory practices [6]. Furthermore, in Canada the Edinburgh Postnatal Depression Scale (EPDS) is widely used to screen for PPD despite the fact that it may not have cultural congruence for all ethnocultural groups [16]. There is a clear need to integrate this existing evidence base. Evolving from foundational research that we have conducted internation-ally, nationally and provincially, our review will establish the veracity of suggestions that existing mental health services might not provide appropriate support to women with PPD [13,14,17]. The complex factors that affect the lives of immigrant women and their preferences for postpartum health care and treatment need to be understood before appropriate measures can be taken to correct the current inability to reach these women [18,19]. The contextual factors and social determinants of health that influence marginalized women’s behavior in accessing existing mental health services have, to some extent, been addressed by the scientific community [20]; however, our review will produce a coherent and comprehensive perspective.

## Methods/design

### Study aim and objectives

Our systematic review with narrative synthesis of reports (peer- and non-peer reviewed) of empirical research aims to provide stakeholders with perspectives on postpartum mental health care services, as experienced by immigrant women, by identifying the acceptability of relevant processes at the individual, community and organizational levels, as these factors are recognized to be critical determinants of effectiveness of services and patient/client outcomes. We will identify specific critical points in care delivery, providing tailored solutions for policy and practice changes. To reach this aim we are using what the Canadian Institutes of Health Research considers integrated knowledge translation (IKT), thus partnering with key stakeholders (integrated knowledge users (IKUs)), as initiated during the establishment of the research questions and early planning for dissemination, and as planned for the entire project duration, when addressing the following objectives:

1. To identify, appraise and synthesize qualitative, quantitative and mixed-methodological empirical studies on the topic;

2. To identify additional knowledge users and mechanisms of KT; and

3. To share our findings through strategic end-of-grant KT.

Ultimately, we will establish the current knowledge base and generate important recommendations for future policy and practice/programming, thus mapping out pathways to health equity.

### Research question

What are the ethnoculturally defined patterns of help-seeking behaviors and decision-making and other predictive factors for therapeutic mental health care access and outcomes with respect to postpartum depression for immigrant women in Canada?

### Population of interest and definitions

This study will review literature and other documents which report on immigrants in Canada, which we define as ‘a person who has settled permanently in another country (Canada)’ [21] but also include economic migrants and skilled workers, temporary foreign workers, documented and undocumented residents, refugee claimants, refugees, asylum seekers and students [22].

#### Postpartum depression

We utilize the Canadian Mental Health Association’s definition of PPD [23], ‘Postpartum depression is more debilitating than the ‘blues’. Women with this condition suffer despondency, tearfulness, feelings of inadequacy, guilt, anxiety, irritability and fatigue. Physical symptoms include headaches, numbness, chest pain and hyperventilation. A woman with postpartum depression may regard her child with ambivalence, negativity or disinterest. An adverse effect on the bonding between mother and child may result. Because this syndrome is still poorly defined and under studied, it tends to be under reported. Estimates of its occurrence range from 3% to 20% of births. The depression can begin at any time between delivery and six months post-birth and may last up to several months or even a year.’

### Study design

Our preliminary search of Canadian literature using Medline failed to identify a knowledge synthesis on the topic and suggested that there would be sufficient literature to inform a systematic review. The lack of an available synthesis of this complex issue, together with the challenge of interpreting multiple forms of literature, makes our narrative synthesis (suitable for integrating qualitative and quantitative research findings) important as a mechanism for drawing together messages from research to guide policy and practice in meaningful ways. This study incorporates two approaches which align with our objectives:

1. Systematic review employing a narrative synthesis to identify, appraise and synthesize reports (peer-reviewed and non-peer reviewed) on empirical research using qualitative, quantitative and mixed-methodological designs;

2. Integrated knowledge translation by partnering with stakeholders throughout the study and during end-of-grant KT, to ensure topic relevancy and to generate and disseminate important recommendations for future policy and practice/programming.

### Search strategies and selection of studies

The search and selection strategies draw upon established systematic review methodologies as outlined by the Centre for Reviews and Dissemination [24] and also incorporate guidelines for selection, appraisal and review of gray literature [25,26]. Gray literature is a ‘field in library and information science that deals with the production, distribution, and access to multiple document types produced on all levels of government, academics, business, and organization in electronic and print formats not controlled by commercial publishing, i.e. where publishing is not the primary activity of the producing body’ [27]. Producers of gray literature report that policy makers are their primary audience and three of the most important topic areas are access to health care, maternal and child health, and minority health [26].

**Figure 1:**
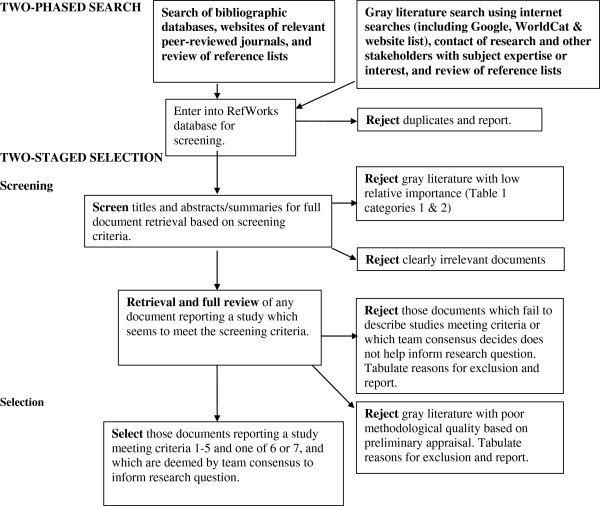
**Search and selection process.** Two search phases will be conducted to provide evidence to contribute jointly to the narrative synthesis. The database search and the gray literature search will be performed concurrently, with the results of both being selected based on the screening checklist. Gray literature will be rejected at the screening stage if it is not considered to be of sufficient relative importance (categories 1 and 2 in Table 1) as determined by AcademyHealth [26]. Gray literature reports describing empirical research will be placed in the narrative synthesis if deemed to have sufficient quality (Category A as per McGrath *et al*. [25].

Two search phases will be conducted (Figure 1). The first will primarily consist of searching electronic databases and websites of relevant journals to identify empirical papers (primary research using working hypotheses (or research questions), which are tested using observations or experimentation) [28] published in peer-reviewed journals. The reference lists of reviews and included studies will also be reviewed for relevant literature. An information scientist (health research librarian) has designed the database search strategies which will be reviewed by the entire research team including IKUs prior to implementation. The following databases will be searched: Ovid MEDLINE In-Process and Other Non-Indexed Citations, Ovid MEDLINE Daily, Ovid MEDLINE 1950-present, Ovid PsycINFO 1987-, Ovid EMBASE 1980-, EbscoHOST CINAHL 1937-, ISI Web of Knowledge Social Sciences Citation Index 1898-, ISI Web of Knowledge Sciences Citation Index 1899-, Scopus 1960-, and CSA Sociological Abstracts 1952-. The search strategy for Ovid MEDLINE [see Additional file 1] will be revised to use subject headings that are appropriate for the other databases. All searches will be implemented with results stored in RefWorks for screening and later retrieval. The second search phase will target gray literature and include select database searches (ISI Web of Knowledge Conference Proceedings Citation Index-Science 1990-, ISI Web of Knowledge Conference Proceedings Citation Index-Social Science and Humanities 1990-, ProQuest Dissertations and Theses), internet-based searches [see Additional file 2], review of reference lists and email or phone contact with researchers and other stakeholders having subject expertise or interest. The entire team will engage in gray literature searching after collaboratively allocating websites to members and adequate training by the information scientist on searching strategies.

Screening and selection of all literature from both search phases will proceed in two stages (Figure 1), using the inclusion criteria contained in a checklist which will be piloted by two reviewers with the first 10% of the database ‘hits’ (Table 1). The screening stage will identify clearly irrelevant literature as well as items for further retrieval (thus meeting all or most of the inclusion criteria) which will be read in full for the selection stage. Selection will be based on two independent reviewers’ agreement, and final team consensus where necessary, of the study’s relevance to the topic and research question. The reasons for exclusion after the screening stage will be tabulated and reported. Study populations which are described solely as ‘ethnic minorities’ without mention of immigrant status or country of birth will not be considered due to ambiguity; all selected literature will have findings (as primary outcomes or after subgroup analysis) directly related to immigrant women. All descriptive and analytical research study designs [29] may be included. Reviews, commentaries and expert opinion papers will not be selected, although the reference lists of reviews will be reviewed for possible identification of further literature. Additionally, gray literature will be rejected at the screening stage if it is deemed to have insufficient quality (Category B or C as per McGrath *et al*. [25]) or not considered to be of sufficient relative importance (categories 1 and 2 in Table 2) as determined by the AcademyHealth advisory committee when defining the scope of gray literature in health services research/health policy for the National Information Center on Health Services Research and Health Care Technology at the National Library of Medicine [26]. Gray literature passing the preliminary quality assessment will present a clear research question(s), state key findings and provide sufficient details on population(s) studied, interventions (if applicable), study design and method of analysis [25]. One of the IKUs, Mrs. Alexandre, is fluent in French and has numerous contacts through which we will hire a casual research assistant to translate publications written solely in French.

**Table 1:** Screening and selection criteria checklist

**Citation** _____________________________	**Yes**	**No**	**Can’t say**
**Criteria for selection**			
1. Publication date 2000 to 2013			
2. English or French language			
3. Empirical research and findings			
4. Study participants live in Canada			
5. Study participants are immigrant			
women (where there is mixed sample			
of immigrant and non-immigrant women			
each paper must have findings specific			
to immigrant women)			
6. Is related to ethnoculturally defined			
patterns of help-seeking behaviors and			
decision-making or predictive factors			
related to postpartum depression			
7. Is related to therapeutic mental health			
care access or outcomes with respect to			
postpartum depression			

The first five items and one of the sixth or seventh must be met for retention.

**Table 2:** **Relative importance of gray literature as used by AcademyHealth**[26]

**5**	**4**	**3**	**2**	**1**
Working papers	Data evaluations	Speeches	Newsletters	Pamphlets
Committee reports	Foundation reports	Annual reports	Biographies	Protocols
Testimony	Government reports	Presentations	Bulletins	Guidelines
Conference proceedings	Grantee publications	Grantee reports	PowerPoint presentations	Poster sessions
	Non-commercially published conference papers	Webcasts	Foundation financial statements	Meeting agendas
	Reports	Theses		Translations
	Special reports	Technical specifications and standards		

Items in columns three to five will be considered for inclusion since they reflect a high relative importance to policy makers and other users of gray literature.

### Quality appraisal

For quality assessment of the identified studies, we will utilize the appraisal tools produced by the Centre for Evidence Based Management [30] which are applicable for qualitative and various (that is, controlled, case study, case–control, survey, and cohort) quantitative research methodologies. These tools were selected because they enable an epistemological, theoretical and technical appraisal as advocated by the Cochrane Collaboration [31]. Reflecting relative expertise, GH will be the primary appraiser for the qualitative studies and JO and MM will be the primary appraisers for quantitative studies. Studies will not be excluded based on quality, although there will be consideration and, where applicable, discussion of their relative contribution to the synthesis.

### Data extraction

The review team will collectively decide on data variables (for example study design, sample population) to include in a data extraction checklist and summary table of the studies, bearing in mind those categories suggested as informative for textual descriptions within the narrative synthesis (Table 3; also refer to Element 2 described in the section Narrative Synthesis). One reviewer will extract the data and one or more team members will review the process, using at least half of the studies, for accuracy and completeness.

**Table 3:** **Tools of likely use for elements 2 through 4 of the narrative synthesis**[24,32]

**Element 2. Preliminary synthesis of findings**	
1. Textual description of the studies	A descriptive paragraph with headings Setting, Participants, Aim, Sampling and Recruitment, Method, Analysis, Results, ‘Thick’ or ‘Thin’ study. This may be represented in tabular format.
2. Groupings and clustering of studies	The data extracted for the textual description will allow papers to be grouped and thus enable patterns between and within studies to be identified. This will be informed by the research questions. Groupings may be by a particular feature, for example, location, method, ethnic groups, form of analysis or main findings.
3. Translating data: thematic analysis	To identify main or recurrent themes in findings.
**Element 3. Exploring relationships within and between studies**	
1. Moderator variables and subgroup analysis	Identifying study characteristics that vary between studies or sample (subgroup) characteristics which might help explain differences in findings.
2. Ideas webbing and concept mapping	Ideas webbing conceptualizes and explores connections among the findings reported in the review studies and often takes the form of a spider diagram.
	Concept mapping links multiple pieces of information from individual studies using diagrams and flow charts to construct a model with relevant key themes.
3. Qualitative case descriptions	Descriptions of outliers or exemplars of why particular results were found in the outcome studies.
**Element 4. Assessing the robustness of the synthesis**	
1. Critical reflection	Summary discussion with the topics of: a) methodology of the synthesis (focusing on the limitations and their possible impact on the results); b) evidence used (quality, reliability, validity and generalizability); c) assumptions made; d) discrepancies and uncertainties identified and how discrepancies were dealt with; e) areas where the evidence is weak or non-existent; f) possible areas for future research and, finally; g) a discussion of the evidence presented that will consider the ‘thick’ and ‘thin’ evidence and comment on similarities and/or differences between evidence.

### Narrative synthesis

Our synthesis will be guided by Popay *et al*. [32] approach to narrative synthesis, which is defined as ‘an approach to the systematic review and synthesis of findings from multiple studies that relies primarily on the use of words and text to summarize and explain the findings of the synthesis’ [32, p5]. The approach is equally suitable for both quantitative and qualitative studies as the emphasis is on an interpretive synthesis of the findings of research rather than on performing a meta-data analysis. It will allow us to encompass the cross-disciplinary and methodologically pluralistic natures of research in this topic area of experiences of postpartum depression of immigrant women in Canada.

The general framework for a narrative synthesis comprises four main elements: 1) developing a theory of how the intervention works, why and for whom; 2) developing a preliminary synthesis of findings of included studies; 3) exploring relationships in the data; and 4) assessing the robustness of the synthesis. Although represented linearly, the processes are not necessarily independent of each other and the synthesis often takes an iterative approach. Within each element there are a variety of tools and techniques which may be employed depending on the nature of the research evidence. Additionally, more tools and techniques may be utilized where appropriate [24]. For instance, since this synthesis will very likely not exclusively synthesize intervention studies, a framework of PPD experiences including healthcare accessibility and outcomes will be created rather than a theory as suggested for Element 1. Table 3 lists the tools which the team envisions using for this synthesis.

#### Element 1: developing a theory

A theory will not play a large role in this synthesis which aims largely to explore experiences of immigrant women rather than any intervention with measurable endpoints and outcome measures. As stated above, after reading the studies we will develop a preliminary framework of immigrant women’s maternity care experiences and outcomes which we will use to interpret and understand our synthesis. This will likely be an iterative process with multiple revisions as we work through Elements 2 through 4.

#### Element 2: developing a preliminary synthesis

The preliminary synthesis will provide an initial description and mapping of the results of all included research studies. The synthesis will be further evaluated by the entire team to identify contextual and methodological factors that have influenced the published results. Interrogation of the preliminary synthesis will facilitate construction of explanations of the ethnoculturally defined patterns of help-seeking behaviors, decision making and therapeutic mental health care access and outcomes with respect to PPD, particularly as to how and why maternity services may have been implemented or have impacted in a particular manner on immigrant women of child-bearing age. Through careful organization of results, cross-literary comparisons will be facilitated by identification of patterns regarding both experiences of PPD, and development and implementation of culturally congruent health services for prevention, promotion and treatment of PPD. For the textual descriptions of the studies (Table 3), we have adapted the assessment of ‘thick’ or ‘thin’ papers using Roen *et al*. work [33] in a systematic review of the evidence about the implementation of the interventions to reduce unintentional injuries among children, which itself drew on the work of Denzin *et al*. [34] and may apply to both quantitative and qualitative findings as the emphasis is on textual (narrative) analysis of the findings. This approach distinguishes between papers that offer greater explanatory insights into their outcome of interest from those that offer a limited insight. Thick papers: a) provide a clear account of the process by which their findings were produced, including the sample, its selection and size with any limitations or bias noted, clear methods of analysis and adjustment for confounding in statistical studies; and b) present a developed and plausible interpretation of the analysis based on the data presented. Alternatively, thin papers: a) lack a clear account of the process by which their findings were produced; and b) present an under-developed and weak interpretation of the analysis based on the data presented.

After creating the textual descriptions, groupings and clusterings of the studies will be undertaken, informed by the research questions. Thereafter, translating the data using thematic analysis will help identify main themes in the findings. For this portion of the synthesis, we will use ATLAS.ti data analysis software (ATLAS.ti Scientific Software Development GmbH, Berlin, Germany) with which the team lead has experience. Publications in Adobe Portable Document Folder (PDF) format will be uploaded as primary documents and coded.

#### Element 3: exploring relationships within and between studies

Patterns emerging from cross-literary comparisons will be subjected to further rigorous evaluation to identify factors that may explain differences, including variances in women’s experiences (including decision-making and help-seeking behaviors), the effects of interventions and implementation of prevention (including screening) and promotion services. We will evaluate the relationships between study characteristics and reported findings and the ways in which these relationships may correspond with key aspects reported in other literature. Careful attention will be paid to the heterogeneity of research methods, methodologies and population characteristics encompassed in the literature through the application of narrative methods, which are particularly suited to synthesizing such findings. Narrative methods facilitate understandings and acknowledgement of the broader influences of theoretical and contextual variables, such as race, gender, socioeconomic status and geographical location. They also enable understanding about the shaping of differences between reported outcomes and study designs related to childbearing populations and the development and implementation of maternity services and health interventions across diverse settings. Key tools used here are moderator variables and subgroup analysis, ideas webbing and concept mapping and qualitative case descriptions (Table 3).

#### Element 4: assessing the robustness of the synthesis

Key to ensuring the robustness of the synthesis is the methodological quality of key literature and the analytical methods used to develop the narrative synthesis. Popay *et al*. recommend that a summary discussion of the synthesis should be provided to reflect on the a) methodology of the synthesis – especially focusing on the limitations and their possible impact on the results, b) evidence used, in terms of quality, reliability, validity and generalizability (for quantitative papers), with an emphasis on possible sources of bias (for qualitative papers we will apply Lincoln and Guba’s [35] principles of confirmability, transferability, credibility, and dependability), c) assumptions made, d) discrepancies and uncertainties identified and how discrepancies were dealt with, and e) areas where the evidence is weak or non-existent, with identification of possible areas for future research [32].

### Integrated knowledge translation

Successful research projects with respect to dissemination are those which respond to a topical issue, have national significance and involve commitment from a team including experienced investigators working in a supportive environment [36]. IKUs from various sectors are involved in this project to ensure that the research is of contextual relevance for current policy, practice and programming needs in Canada. The organizations which are represented include Citizenship and Immigration Canada (CIC), Public Health Agency of Canada (PHAC), Alberta Health Services (AHS) and the Multicultural Health Brokers Co-operative (MHBC). The relevance of this project to each organization and IKUs role was described in our previous protocol for another knowledge synthesis project [37]. In addition to using IKUs, a systematic review expert (MM) will help ensure rigor and validity of the findings and recommendations.

Concern with immigrant maternity experiences is emerging within Canada [38] as indicated by recent national guidelines from the PHAC explicitly referring to population diversity and the need to tailor services to the needs of those they serve [39]. Moreover, voluntary sector immigrant agencies are becoming active in several cities (for example, the MHBC in Edmonton) to support immigrant women with their access to maternity services. A rapidly changing demography and observations from maternity care providers throughout AHS, coupled with the known health disparities that immigrant women experience, present many challenges on the trajectory of maternity care provision whether in the community or hospital.

### Partnership interactions

**Figure 2:**
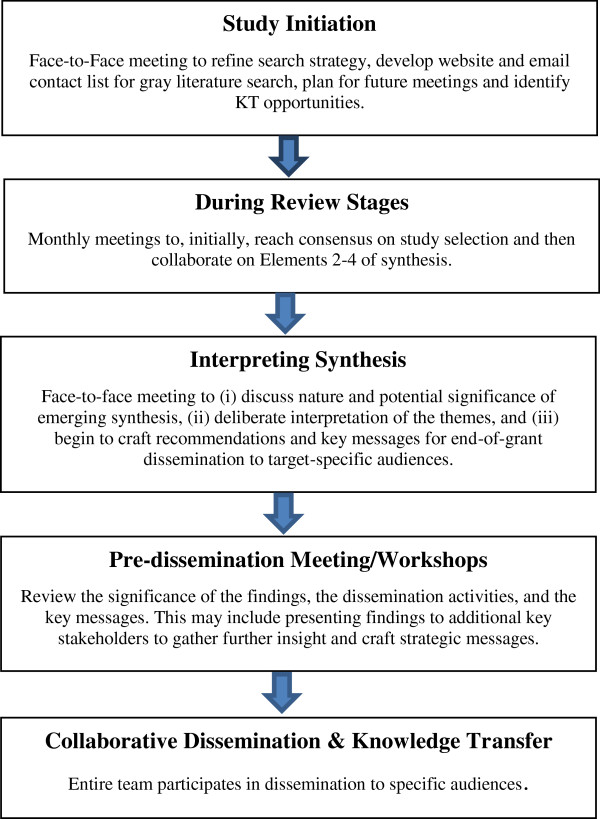
Team interactions during the project.

Our partnerships with key stakeholders and IKUs were established over several years through collaborations within the first author’s program of research associated with a Canada Research Chair in Ethnicity and Health. All team members contributed to the development of the review question to facilitate practice, policy and academic relevance. The interactions between the team during the operationalization of the review are illustrated in Figure 2.

### Dissemination of findings and recommendations

Our dissemination goal is to ensure key messages are delivered in audience-specific manners, aligning with the needs of IKUs, to optimize their impact on policy and practice change throughout the health service, public health, immigration and community sectors. The specific KT strategies can be categorized by the target audience.

For mixed-audience KT, a research briefing paper for health practitioners and policy/decision makers in Canada and an accessible plain language fact sheet will be professionally designed and produced, with wide dissemination using the activities and links of the academic team members and IKUs. The fact sheets, particularly, will facilitate transfer of messages to the public as well as healthcare professionals, policy-makers and other knowledge users. Contribution to academic theory and practice will occur through publication of findings in accessible international journals. The IKUs will be invited to co-author publications. Given that the research team involves individuals who are engaged in community and hospital-based health services with immigrant women, knowledge translation has begun and will continue through to public dissemination via community meetings with families, women’s groups and workshops related to refugee/immigrant mental health. Likewise, additional knowledge users (multi-provincial) will be invited to attend community-based seminars/workshops. Publicizing findings at multicultural and immigration/multicultural events will target community, provincial and national leaders. IKUs YC and ME will be primary agents for these activities.

Diffusion and knowledge transfer mechanisms will inform policy and practice and include: international, national and regional networks and conferences attended by policy makers, such as the Qualitative Research for Policy Making Conference (UK), a multi-provincial E-seminar, and a provincial email knowledge transfer network, Collaborative Research in Ethnicity Social Care and Health, created and convened by the lead reviewer (GH). Presentations will be made by the IKUs when they attend conferences and advisory committee meetings with key federal and provincial policy makers and ministers.

An E-seminar will be developed with presentations by the principal investigator (GH) and IKUs to disseminate the findings to health care practitioners, and additional workshops/seminars may be facilitated at national health research conferences (for example, for neonatal/obstetrical or mental health nursing). IKUs JF and MY will help integrate findings into professional practice via their clinical and diversity education capacities.

## Discussion

This synthesis seeks to address gaps in knowledge and generate new interpretations of research findings and other literature for translation into improvements to maternity services provision throughout Canada. It aims to have substantive and sustainable impact on health outcomes, practice, programs and/or policy in the study context. The project does not directly evaluate interventions/impacts on service delivery but will provide an important basis to guide such developments and ensure their acceptability and effectiveness for immigrants. Programs funded through the PHAC and CIC may thus be enhanced to optimize outcomes. The work will have relevancy to other practice, programs and/or policy contexts. The project involves conducting a narrative synthesis of quantitative and qualitative research. This will be important methodologically as there is a recognized need to bring together comprehensive evidence on research and policy questions to inform programs and service delivery but few examples of the integration of these types of research exist. The findings of the synthesis will have direct relevance in guiding the provision of maternity services for immigrant groups. In addition, the synthesis is likely to identify themes that have wider application for service delivery (including healthcare, immigration and multiculturalism) and public health initiatives/programs in relation to immigrant groups. It will thus assist in responding to wider issues of service provision and delivery. This includes the value of the synthesis in terms of the education and training of health professionals and settlement workers in achieving cultural competence.

## Abbreviations

AHS: Alberta health services; CIC: Citizenship and immigration Canada; IKT: Integrated knowledge translation; IKU: Integrated knowledge users; KT: Knowledge translation; MHBC: Multicultural health brokers co-operative; PHAC: Public health agency of Canada; PPD: Postpartum depression.

## Competing interests

The authors declare they have no competing interests.

## Authors’ contributions

GH conceptualized the study and prepared the draft of the research proposal. MM provided insight to the methodological approach of the narrative synthesis and quality appraisal. MM, JO, YC, ME, DK, MY and JF contributed to the proposal development as related to research questions, aim/objectives and dissemination activities. All authors assisted with manuscript preparation. All authors read and approved the final manuscript.

## Authors’ information

GH: http://www.chairs-chaires.gc.ca/ and http://www.cresh.ualberta.ca/.

## Supplementary Material

Additional file 1 Search strategy for MEDLINE. Click here for file

Additional file 2 List of websites generated for searching gray literature. Click here for file
