# Supplementary material for: Immigrant women’s experiences of postpartum depression in Canada: a protocol for systematic review using a narrative synthesis
Source: Syst Rev. 2013 Aug 21;2:65. doi: 10.1186/2046-4053-2-65 (PMC3765819; doi:10.1186/2046-4053-2-65)
Supplement: Additional file 1 — Search strategy for MEDLINE. [file 2046-4053-2-65-S1.docx]

**Additional File 1. Search strategy for MEDLINE.**

1. Depression, Postpartum/

2. Puerperal Disorders/

3. Depression/

4. 2 and 3

5. ((postnatal or post natal or post-partum or postpartum or maternal or parental or puerper*) adj3 depress*).mp.

6. 1 or 4 or 5

7. "emigrants and immigrants"/ or refugees/ or "transients and migrants"/

8. (immigrat* or immigrant* or refugee* or newcomer* or new-comer* or alien* or adoptive citizen* or foreigner* or incomer* or naturalized citizen* or foreign born or country of birth or migrant worker* or foreign worker*).tw.

9. ((international or foreign) adj2 student*).tw.

10. 7 or 8 or 9

11. 6 and 10

12. acculturation/ or circumcision, female/ or cross-cultural comparison/ or cultural characteristics/ or cultural diversity/

13. Cultural Competency/ or Prejudice/ or "Cultural Diversity"/ or "Transcultural Nursing"/

14. (cultural* competen* or cultur* appropriate or prejudice* or racist* or racism or bigot*).mp.

15. 12 or 13 or 14

16. 6 and 15

17. 11 or 16

18. exp canada/

19. canada.cp.

20. (canada or canadian$ or alberta or british columbia or columbie britannique).af.

21. (saskatchewan or manitoba or ontario or quebec or new brunswick or nouveau brunswick).af.

22. (nova scotia or nouvelle ecosse or prince edward island or ile du prince edward or newfoundland or terre neuve or labrador or nun?v?t or nun?v?t or nwt or territoires du nord ouest or northwest territories or yukon).af.

23. (canada or canadian$ or alberta or british columbia or columbie britannique).in,jw,nw,jx.

24. (saskatchewan or manitoba or ontario or quebec or new brunswick or nouveau brunswick).in,jw,nw,jx.

25. (nova scotia or nouvelle ecosse or prince edward island or ile du prince edward or newfoundland or labrador or nun?v?t or nwt or northwest territories or territoires du nord ouest or yukon).in,jw,nw,jx.

26. or/18-25

27. 17 and 26
