# Supplementary material for: Immigrant women’s experiences of postpartum depression in Canada: a protocol for systematic review using a narrative synthesis
Source: Syst Rev. 2013 Aug 21;2:65. doi: 10.1186/2046-4053-2-65 (PMC3765819; doi:10.1186/2046-4053-2-65)
Supplement: Additional file 2 — List of websites generated for searching gray literature. [file 2046-4053-2-65-S2.docx]

**Additional File 2. Some identified websites that will be searched for grey literature.**

| **Canadian Mental Health Association** |
| --- |
| **lien MOMS Link** |
| **Best Start (Ontario's Maternal Newborn and Early Child Development Resource Centre)** |
| **Health Canada (including women’s health contribution program page)** |
| **Public Health Agency of Canada** |
| **Citizenship and Immigration Canada** |
| **Government of Canada’s Policy Research Initiative** |
| **Status of Women in Canada** |
| **Canadian Research Institute for the Advancement of Women** |
| **New York Academy of Medicine Grey Literature Collection** |
| **Canadian Public Health Association** |
| **American Public Health Association** |
| **Agency of Healthcare Research and Quality** |
| **National Center for Education in Maternal and Child Health** |
| **Institute for Women’s Policy Research** |
| **Access Alliance** |
| **Nursing Health Services Research Unit** |
| **Centre of Excellence for Research on Immigration and Settlement** |
| **Ontario Women’s Health Network** |
| **Wellesley Institute** |
| **Canadian Policy Research Networks** |
| **Ontario Healthy Communities Coalition** |
| **Mental Health Commission of Canada** |
| **Centre for Addiction and Mental Health** |
| **PsycEXTRA** |
| **Virtual Library for Public Health** |
| **GreySource** |
| **Metropolis.net** |
| **Women’s Health Research Foundation of Canada** |
| **BC Centre of Excellence for Women’s Health (Publications and Data Directory under BCCEWH initiatives)** |
| **BC Provincial Health Services Authority** |
| **Atlantic Centre of Excellence for Women’s Health** |
| **Prairie Women’s Health Centre of Excellence** |
| **Canadian Women’s Health Network** [**http://www.cwhn.ca/**](http://www.cwhn.ca/) |
| **Women and Health Care Reform** [**http://www.womenandhealthcarereform.ca/**](http://www.womenandhealthcarereform.ca/) |
| **Feelings in Pregnancy** [**http://www.feelingsinpregnancy.ca/**](http://www.feelingsinpregnancy.ca/) |
| **Saskatchewan Maternal Mental Health** |
| **Women’s Health Data Directory** |
| **The Survey/Le Sondage** |
| **Canadian Foundation for Women’s Health** |
| **Immigrant Women’s Health Centre** |
| **Immigrant Women’s Centre** |
| **Canadian Association of Midwives** |
| **Alberta Health and Wellness** |
| **British Columbia Centre for Health Services and Policy Research** |
| **Institute for Clinical and Evaluative Sciences (ICES): Ontario’s health services and utilization research agency** |
| **Place, Migration and Health** |
| **Canadian Health Services Research Foundation** |
| **UBC Centre for Health Services and Policy Research** |
| **Canadian Institute for Health Information** |
